# Editorial: Photosynthetic and photorespiratory organelles: metabolism, dynamics, and signaling

**DOI:** 10.3389/fpls.2023.1293246

**Published:** 2023-10-10

**Authors:** Jianping Hu, Ronghui Pan, Andreas P. M. Weber, James Whelan

**Affiliations:** ^1^ Michigan State University-Department of Energy Plant Research Laboratory, Michigan State University, East Lansing, MI, United States; ^2^ College of Agriculture and Biotechnology, Zhejiang University, Hangzhou, China; ^3^ Institute of Plant Biochemistry, Cluster of Excellence on Plant Science (CEPLAS), Heinrich-Heine-University, Düsseldorf, Germany; ^4^ College of Life Science, Zhejiang University, Hangzhou, China

**Keywords:** peroxisomes, mitochondria, chloroplasts, organelle targeting, chlorophagy, retrograde signaling, photorespiration, biotic stress response

## Introduction

Plastids (chloroplasts), mitochondria, and peroxisomes are subcellular organelles with essential roles in energy capture, conversion, and metabolism, among many pivotal cellular processes. The biogenesis, protein targeting, proliferation, and degradation of these organelles are highly regulated in the plant cell. Several metabolic pathways, including photosynthesis and photorespiration, and the metabolism of fatty acids and derivatives, are attributed to the functional coordination of these organelles. In addition, plastids are key hubs for emitting multiple retrograde signals to regulate nuclear gene expression in response to developmental and environmental cues. Emerging evidence also points to the role of these organelles as environmental sensors involved in the balance between plant growth and defense.

This Research Topic consists of 4 original research articles and 4 review articles that highlight recent advances in the understanding of the dynamics and the metabolic and signaling functions of the three energy organelles, as well as inter-organellar crosstalk and networking. How to improve crop yield through regulating photosynthetic assimilate distribution and source-sink balance is also discussed.

## Organelle dynamics

The proper function of the photosynthetic and photorespiratory organelles requires a tight control of their protein homeostasis, which is governed by processes from organelle biogenesis, protein targeting, to protein and organelle degradation.

Gill-Hille et al. tested the organelle targeting of several Arabidopsis orthologs of the components of the Presequence Translocase-Associated Motor (PAM) complex, which drives the translocation of proteins across the mitochondrial inner membrane. Interestingly, Pam18 orthologs, as well as the PAM complex-associated proteins Tim15 and Mge1, are dual targeted to mitochondria and plastids. These results are consistent with the collaborative function of mitochondria and plastids and the fact that proteins destined to both of these two organelles utilize N-terminal cleavable targeting peptides, suggesting the dynamic nature of protein import components and mechanisms.

The study of chlorophagy, the selective autophagy-dependent turnover of chloroplast proteins in response to developmental and environmental cues, is still in its infancy. The review by Wan and Ling provided a comprehensive overview of several chlorophagy pathways identified in plants and pointed out the many remaining knowledge gaps, especially those of the signals and organellar receptors in these pathways.

## Chloroplast-nucleus signaling

The complexity and diversity of plastids, combined with the tissue/cell-specific and developmental nature of plastid signaling, makes it a challenge to fully understand the anterograde and retrograde signaling pathways involving plastids. Jan et al. summarized the current knowledge of multiple plastid retrograde pathways, primarily best understood in chloroplasts. Chloroplast biogenesis requires the depression of a variety of factors upon perception of light that coordinate not only organelle and nuclear transcription, but also protein translation and organelle assembly and degradation. While several pathways are known, how the molecular components that mediate these pathways are integrated with the metabolite signaling molecules remains to be resolved.

While the impact of abiotic stress on chloroplast retrograde signaling has been intensively studied, less is known about how biotic stresses can affect chloroplast signaling. Virus infection in plants is known to impact chloroplast function, directly and indirectly. A study in *Nicotiana benthamiana* by Ershova et al. revealed that infection with potato virus X (PVX) induces a decrease in transcript abundance for several markers of chloroplast signaling. A Kunitz peptidase inhibitor-like protein (KPILP), which is induced upon PVX infection, plays a role in downregulating the expression of chloroplast retrograde signaling genes, reducing photoassimilate accumulation, and controlling plasmodesmata permeability, thus linking viral infection to chloroplast signaling.

## New metabolic function and organellar network revealed by omics studies

Using bioinformatics followed by fluorescence microscopy, Zhang et al. identified 4 new Arabidopsis peroxisomal members of the short-chain dehydrogenase/reductase (SDR) superfamily of NAD(P)(H)-dependent oxidoreductases. Phylogenetic analysis of SDR orthologs in diverse plant species and *in silico* expression profiling of the Arabidopsis genes suggested the possible involvement of these new SDRs in reproduction and seed germination. This study provides a foundation for further investigation of organellar redox control during plant development.

Castor bean seeds contain large amounts of storage oil in the endosperm tissue surrounding the cotyledons. 90% of castor bean oil consists of a rare, commercially important hydroxylated fatty acid, ricinoleic acid. How this hydroxylated fatty acid is degraded is to date unknown. Wrobel et al. reported a proteomic analysis of plastids, mitochondria, and peroxisomes from germinating castor bean seeds. Based on the proteomic data, they reconstructed the metabolic network operating during storage oil mobilization and discovered that conversion of ricinoleic acid to acetyl-CoA during ß-oxidation involves the peroxisomal Δ^3^,Δ^2^-enoyl-CoA isomerase and one or several α-2-hydroxy acid oxidases. They further showed that endosperm mitochondria use amino acids as the predominant substrate for respiration and that Rubisco is involved in providing pyruvate for plastid-localized fatty acid biosynthesis.

## Role of photorespiration in biotic stress response

To defend themselves against biotic stresses, plants are equipped with a complex, multi-tiered immune system that requires the coordination of various biological and metabolic pathways. Emerging evidence have demonstrated a strong role of photorespiration, a byproduct pathway of oxygenic photosynthesis that spans chloroplasts, peroxisomes, mitochondria, and the cytol, in plant immunity. The review by Jiang et al. discussed the role of hydrogen peroxide (H_2_O_2_), a crucial product of photorespiration, as well as the likely participation of photorespiratory metabolites and other molecules in the plant immune response network. Understanding the mechanistic links between photorespiration and plant immunity will be instrumental to crop engineering to improve photosynthesis while reducing possible negative impacts on plant immunity.

## Regulating photosynthetic assimilate allocation for cotton yield improvement

To grow robustly, it is critical for plants to coordinate the source and sink partitioning of photosynthetic assimilates. In their review, Qin et al. highlighted mechanisms in photosynthetic assimilate transport and partitioning and the source-sink balance in the context of improving cotton yield. As a global cash crop, the determinate growth and multiple fruiting of cotton make the coordination between source and sink particularly important. Since chloroplasts, mitochondria and peroxisomes are key sites for photosynthesis-driven carbon metabolism, it would be interesting to assess their importance in regulating carbon partition to ultimately contribute to crop yield.

## Author contributions

JH: Writing – original draft, Writing – review & editing. RP: Writing – original draft. AW: Writing – original draft. JW: Writing – original draft.

